# A Sporulation-Specific sRNA Bvs196 Contributing to the Developing Spore in *Bacillus velezensis*

**DOI:** 10.3390/microorganisms10051015

**Published:** 2022-05-12

**Authors:** Tingting Xu, Xiangying Li, Kerong Chen, Haoxin Qin, Zhengkai Yi, Yuan Meng, Zhenyu Liu

**Affiliations:** College of Plant Protection, Shandong Agricultural University, Taian 271018, China; tingtingdan@163.com (T.X.); nongdaxiangyingli@126.com (X.L.); 18864820763@163.com (K.C.); 18754802853@163.com (H.Q.); 13176908815@163.com (Z.Y.); mengyuan20210226@163.com (Y.M.)

**Keywords:** *Bacillus velezensis* PEBA20, sRNA, sporulation, Bvs196

## Abstract

Many putative sRNAs have been characterized using bioinformatic analysis and high-throughput sequencing in Gram-positive *Bacillus* strains, but there are only a few functional studies on the sRNAs involved in the spore formation developmental process. In particular, there is no sRNA confirmed experimentally to regulate the late stages of sporulation. Bvs196 is an sRNA with a length of 294 nucleotides that is abundantly expressed in the stationary phase of several media and independently transcribed in *Bacillus velezensis* strain PEBA20, as validated by RNA-seq and Northern blot,. It is also confirmed, by qRT-PCR, that Bvs196 is transcribed abundantly throughout the intermediate and late stages of sporulation. Using the *gfpmut3a* gene transcriptional reporter demonstrates that Bvs196 is expressed specifically in the forespore during sporulation and controlled by σ^F^ and σ^G^ (mainly by σ^G^). This was observed by fluorescence microscopy and multi-function microplate reader. Further evolutionary conservation analysis found that Bvs196 is widely present in *Bacillus* with a strongly conserved and stable secondary structure. Resistance phenotypic assays of spores formed from the Bvs196 deletion mutant, the overexpressed Bvs196 mutant, and the wild-type strain revealed that the absence of Bvs196 led to reduced heat and UV resistance and enhanced formaldehyde resistance. We determined, by MST analysis, that Bvs196 can directly interact with *spo0A* and *sspN-tlp* mRNAs in vitro, and that short incomplete complementary paired bases affect the binding affinity of Bvs196 to target mRNAs. Our results suggest that Bvs196 is a novel sporulation-specific sRNA of *B. velezensis*, 294 nt in length, independently transcribed under the control of σ^F^ and σ^G^ in the forespore during sporulation, and that it affects spore resistance, and is able to directly interact with *spo0A* and *sspN-tlp* mRNAs. The remarkable conservation and impressive expression level of Bvs196 imply that it acts as an important conservative regulator, presumably by interacting with many other unknown targets in the forespore, and therefore contributing to spore properties. This work provides new clues for further understanding of the spore formation regulatory network.

## 1. Introduction

*Bacillus velezensis* produces endospores and is very close in evolution to the Gram-positive model bacteria *Bacillus subtilis.* Over the years many studies have been carried out on this species, focusing on its possible utilization in the agricultural industry as a form of effective phytostimulator, biofertilizer, and biocontrol agent for managing plant diseases. *B. velezensis* strains can adapt to a variety of environments, such as soil, food, and aquatic environments, since they are isolated from various ecological niches [1]. In order to survive in changing environments, specifically in extreme conditions, *Bacillus* strains have developed complex regulatory networks during the long-term evolutionary process [2]. Sporulation is a model system which is especially suitable for elucidating transcriptional regulations. Morphological events, asymmetric division, and engulfment are important check points for sporulation, and are highly coordinated by a complex regulatory network which is consists of hundreds of time-specific and compartment-specific sporulation genes [3]. There are two main types of regulatory factors in regulatory networks. One is protein transcription factors which control the genes necessary for survival under specific conditions and mainly regulate the transcription level of genes [4]. The other is small regulatory RNAs (sRNAs) which mainly fine-tune post-transcriptional regulated gene expression by incomplete complementary base-pairing with their target mRNA, change protein translation efficiency, or affect the stability of mRNA [5].

The majority of sRNAs have been described and characterized in great detail in Gram-negative bacteria, especially *Escherichia coli* and *Salmonella enterica* [6,7,8] whereas only a few sRNAs, mainly *B. subtilis*, have been functionally studied in Gram-positive bacteria, [9]. However, only 21 sRNAs were identified and experimentally validated in *B. velezensis* FZB42 [10]. To date, several sRNAs whose expressions are altered upon sporulation have been detected. Silvaggi et al. investigated the expression of sRNAs during sporulation in *B. subtilis* [11], and identified four sRNAs: the first, sRNA SurA, was found to be expressed before asymmetric division; the second, sRNA SurC, was transcribed in the mother cell under the control of σ^K^; the third, a complex pattern of sRNAs in the *polC-ylxS* intergenic region, could transcribe under the control of σ^G^ in the forespore as well as σ^K^ in the mother cell; and the fourth, an additional sRNA from the *yocG-yocH* intergenic region, was transcribed under the control of σ^E^ in the mother cell. Among the four sRNAs, SurC and sRNAs in the *polC-ylxS* intergenic region were conserved in *B. velezensis* [12]. Subsequently, sRNA CsfG, also conserved in *B. velezensis*, was confirmed to have a high expression level in several conditions [13]. The researchers considered CsfG to have an important physiological role in sporulation, as it is controlled by σ^F^ and σ^G^ and restricted in the forespore [14]. However, there are still no mRNA targets identified for CsfG. Additionally, disrupting these sRNAs did not cause any obvious sporulation defect. Cavanagh and Wassarman only confirmed that 6S-1 RNA (also called *bsrA*) contributes to the timing of onset of sporulation in *B. subtilis*, since lack of 6S-1 RNA led to earlier sporulation. In addition, after the commitment to sporulate, lack of 6S-1 RNA has no effect, so it is not considered that 6S-2 RNA affects the timing of sporulation [15]. However, another researcher found the Δ6S-2 RNA exhibits earlier sporulation [16]. These results suggest that the two 6S RNAs in *B. subtilis* together contribute to transcriptome adaptation, thereby improving the adaptability of *B. subtilis* under a variety of environments. The only dual-function sRNA SRI, a *trans*-acting sRNA and, simultaneously, a gene encoding a small protein, SR1P, has recently been shown to be able to reduce translation of *kinA* mRNA (associated with sporulation initiation) without affecting *kinA* mRNA stability in addition to regulating the translation initiation of *ahrC* mRNA by combining with the RNA chaperone CsrA [17,18].

Although the above-mentioned bacterial sRNAs have been detected and play an important role in the cellular process, we believe that there are many more sRNAs to be discovered. Only a few sRNAs have been shown to be involved in the spore-formation process, and the regulatory mechanisms should be revealed. Therefore, it is of great interest to study the functions of the sporulation-specific sRNAs, which also enrich the regulatory network of sporulation. In the study, we found a highly expressed sRNA Bvs196 of interest in *B. velezensis* strain PEBA20 in the stationary phase. sRNA Bvs196 was homologous with a RNA fragment, named S357 in *B. subtilis*, which was considered as a completely independently transcribed *trans*-acting sRNA, controlled by the predicted sporulation-specific sigma factors using promoter cluster analysis [13,19]. However, no experiment has, so far, confirmed the above hypotheses. Therefore, in the study, we confirmed experimentally that Bvs196 is a sporulation-specific sRNA of *B. velezensis*, and revealed the regulatory mechanism of Bvs196 in sporulation by identifying the specific direct targets of bvs196 and the control by sigma factors. 

## 2. Materials and Methods

### 2.1. Strains, Plasmids, and Growth Conditions

All *Bacillus* strains used in this study were derived from our collected laboratory strain *Bacillus velezensis* strain PEBA20 (NCBI Reference Sequence: NZ_CP046145.1) (see Table S1). All *Bacillus* strains and *Escherichia coli* strains (DH5α or JM110) were routinely grown in liquid Luria–Bertani (LB) broth at 28 °C or 37 °C, and the solid plates was LB supplemented with 1.5% agar. When required, ampicillin, erythromycin, and chloramphenicol were added to final concentrations of 100, 1, and 5 μg/mL, respectively. M9 medium and NSM9 medium (almost the same as M9 except the NH_4_Cl is 0.1 g per liter) were prepared as previously described [13].

Sporulation was induced by nutrient exhaustion in Difco sporulation medium (DSM) [20] or by the resuspension method in resuspension medium (RM) [21] at 37 °C.

For growth curves, when cells from a frozen stock formed colonies on the LB plate, a single colony was inoculated into 5 mL liquid LB and grown overnight. It was then 1:100 diluted to 45 mL liquid LB per bottle and cultured at 37 °C with 550 r/min shaking in an automatic microbial growth curve analyzer (Jieling Instrument Manufacturing Co., Ltd., Tianjin, China), and optical density at 600 nm (OD_600_) was monitored every 20 min. 

To quantify sporulation frequency, cells were induced to sporulation in DSM. After being grown for 30 h at 37 °C, the number of colony-forming units (CFU) /mL before and after wet-heat treatment (20 min at 80 °C) was calculated, and the sporulation frequency was calculated as CFUs/mL after heat treatment divided CFUs/mL before heat treatment. 

To quantify fluorescence activity, cells were induced to sporulation by the resuspension method. Fluorescence activity was measured by Synergy2 (BioTek, Winooski, VT, USA) and was reported as RFU (relative fluorescence unit normalized to cell density).

### 2.2. Spore Resistance Assay

Spores of *Bacillus* strains were formed from DSM medium cultured for 60 h at 37 °C with no antibiotics, purified as described previously [22], and finally stored in double-distilled water and protected from light at 4 °C. Resistance assays (resistance to heat, ultraviolet, and formaldehyde treatments) were carried out as described previously [23].

### 2.3. RNA Extraction

Then, 1–2 mL of strain PEBA20 and derivative cell cultures grown under different conditions (LB, M9, NSM9, and RM) were collected by centrifugation at 12,000× *g* for 1 min at 4 °C, and cleaned with RNase-free water and frozen in liquid nitrogen and stored at −80 °C. Total RNA was extracted using TRIzol reagent (Invitrogen, Carlsbad, CA, USA) following the manufacturer’s protocol with a few modifications. Before being treated with 1 mL TRIzol Reagent, samples were resuspended in 200 μL TE buffer and 20 μL lysozyme (200 mg/mL) before incubation for 15 min at 37 °C with vortexing for 30 s every 5 min. The following experimental procedures were the same as the protocols. The concentration of total RNA obtained was measured with NanoDrop (Thermo Fisher Scientific, Waltham, MA, USA).

### 2.4. Generation of Mutants and Overexpression Strains

Mutant strains were constructed as described previously [24]. Above all, the pMAD plasmid was used to construct two new vectors, one of which contained the *gfpmut3a* (*gfp* for short) gene within upstream and downstream sequences of *bvs196*, and the other only contained upstream and downstream sequences of *sigG*. Primers used to generate each DNA fragment are listed in Supplementary Table S2. DNA fragments were ligated into the BamHI and Hind III sites in pMAD by homologous recombination using ClonExpress^®^ MultiS One Step Cloning Kit Vazyme Biotech Co., Ltd., Nanjing, China) [25]. Then vectors were transformed into wt (for *Δbvs196* or *ΔsigG*) or *ΔsigG* (for *Δbvs196ΔsigG*) cells and recombinants identified as those that were sensitive to erythromycin or were detected green fluorescence if necessary. 

The pHT01 vector was used for overexpression strains or complementation strains. The first *Pgrac*-inducible promoter was removed from pHT01 to create a new vector pHTCE, and HindIII and BamHI double digestion was carried out, followed by ligation of the *bvs196* gene by homologous recombination to obtain a new plasmid pHTCE-*bvs196*. The complementary strain or overexpression strain was constructed by transforming pHTCE-*bvs196* into *Δbvs196* or wt, separately, named *cBvs196* or *obvs196*. The stains above were selected with 5 μg/mL chloramphenicol. 

### 2.5. Bioinformatics Analysis

A multiple-sequence alignment among *bvs196* and homologs was performed using LocaRNA with default parameters [26]. The secondary structure was performed using RNAalifold [27] based on the minimum free energy with default settings. The sequence conservation of *bvs196* was conducted via BLASTn with all bacterial genomes in NCBI nr Database (2019.01), with default parameters. Values in accordance with E-value < 1 ×10^−10^, percent identity >50% and query cover >50% were considered conserved. The tree was constructed based on an alignment of the whole genomes using a maximum likelihood (ML) algorithm implemented in PhyML and the GTR (generalized time-reversible) model bootstrapped 100 times via REALPHY [28]. The target genes of Bvs196 were predicted by using three on-line tools, TargetRNA2 [29], CopraRNA 2.1.3 using IntaRNA 2.3.1 [30], and IntaRNA 3.2.0 [31]. The parameter in TargetRNA2 was altered to 100 NTs before the start codon and 100 NTs after the start codon. CopraRNA and IntaRNA 3.2.0 were performed by default settings.

### 2.6. qRT-PCR Analysis

Next, 1 µg total RNA was used to synthesize cDNA using HiScript III RT SuperMix for qPCR (+gDNA wiper) (Vazyme Biotech Co., Ltd., Nanjing, China) following the manufacturer’s protocol. Subsequently, relative quantitative analysis of the expression levels of mRNAs and sRNAs was performed on the Roche LightCycler^®^ 96 system with ChamQ SYBR qPCR Master Mix (Vazyme Biotech Co., Ltd., Nanjing, China). The primer used in qRT-PCR are listed in Supplementary Table S2. The 16S rRNA gene was used as the endogenous reference control to normalize the expression of target genes, and fold changes of target genes was analyzed by the 2^−ΔΔCT^ method. Three biological replicates for each sample were conducted.

### 2.7. Northern Blot

Northern blot analyses were carried out using a DIG Northern Starter kit (Roche, Mannheim, Germany) following the manufacturer’s protocol. The primers used in the Northern blot are listed in Supplementary Table S2. Band intensity was detected using the FluorChem E system (ProteinSimple, San Jose, CA, USA).

### 2.8. Fluorescence Microscopy

Cells harboring the *gfp* reporter were collected at hours 5 or 6 after the onset of sporulation in DSM medium by centrifugation at 8000 rpm for 1 min. Subsequently, cells were resuspended in 0.2 mL Hanks’ balanced salt solution (HBSS) with 5 μg/mL FM4–64 dye (Molecular Probes) to stain membranes, and then spotted on thin 1% agarose pads with coverslips. Fluorescence microscopy was performed using a Zeiss LSM 800 microscope equipped with Axio Observer. Z1/7 and Plan-Apochromat 63x/1.40 Oil DIC M27 objective. Images were visualized using ZEN 3.2 (blue edition) software (Carl Zeiss, Oberkochen, Germany). 

### 2.9. Microscale Thermophoresis (MST) Assay 

MST experiments were carried out for quantitative interaction analysis between sRNAs and the target mRNAs in vitro [32,33]. 5′ end of targets mRNA fragment (*spo0A-99 nt* and *sspN-tlp-70 nt*) were labeled by 6-FAM fluorescent molecules synthesized by BGI (The Beijing Genomics Institute, Beijing, China). Bvs196 (312 nt) and derived mutants were synthesized in vitro using T7 High Yield RNA Transcription Kit (Vazyme). The sequences and primers are listed in Supplementary Table S2. Before binding affinity experiments on Monolith NT.115 (NanoTemper Technologies, Munich, Germany), samples were centrifuged at 13,000 rpm for 10 min, then, the manufacturer’s instructions were followed to prepare the 16 mixed samples using diethylpyrocarbonate water as assay buffer, containing 200 nM labeled mRNA targets and series dilution concentrations of non-labeled sRNAs (from 7.63 nM to 500 μM). The standard capillaries (Cat#MO-K022) were then dipped into each tube from 1 to 16, and subsequently measurement was started at room temperature with auto-detect excitation power and medium MST-power. Data analyses were carried out using Nanotemper MO Affinity Analysis Software.

## 3. Results

### 3.1. Characterization of the sRNA Bvs196

**Figure 1:**
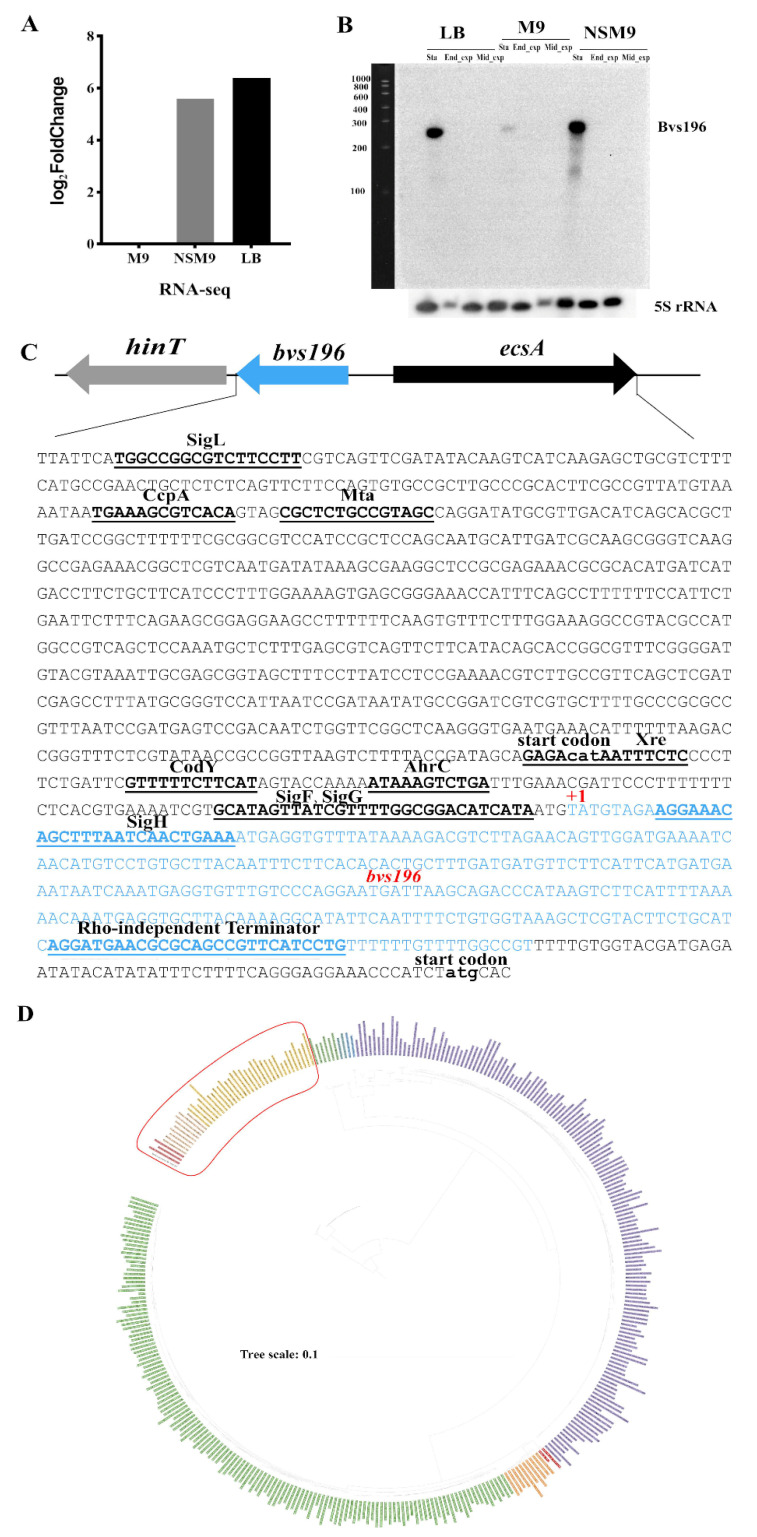
Basic characteristics of Bvs196. (**A**) The expression levels of *bvs196* at the stationary phase in M9, NSM9, and LB compared to M9 medium based on RNA-Seq analysis in *B. velezensis* strain PEBA20. (**B**) Transcript levels of *bvs196* at different times in M9, NSM9, and LB medium detected by Northern blot. Sta, the stationary phase; End_exp, end of the exponential phase; Mid_exp, middle of the exponential phase. (**C**) The genes situated next to *bvs196* and the nucleotide sequence of *bvs196*, all putative regulatory binding sites, were marked. (**D**) Phylogeny tree of 253 *Bacillus* genomes. The tree was generated based on an alignment of the whole genomes using REALPHY. The red frame included strains missing the 3′ ends of the *bvs196* orthologs.

Bvs196 was identified as an sRNA with a predicted length of 294 nucleotides (nt) based on our Illumina RNA-seq data (data have not yet been published), which was significantly upregulated (log2FoldChange = 5.60) in the stationary phase under N-starvation culture medium (NSM9) compared to M9 medium, and was significantly upregulated (log2FoldChange = 6.40) in the stationary phase of LB medium compared with M9 medium (Figure 1A). Northern blot was performed to confirm whether Bvs196 is an sRNA transcribed independently in *B. velezensis* and to determine its size. The results revealed an obvious RNA band, approximately 300 nt in length in NSM9_Sta and LB_Sta, and a light RNA band in M9_Sta, but not in other conditions, indicating that Bvs196 was transcribed independently and was 294 nt long (Figure 1B). The results of Northern blot further verified the reliability of the transcriptome data.

Promoters and transcription factor (TF) binding sites were identified in the DBTBS database [34] using 900 nt upstream sequences of the *bvs196* putative transcription start site. When the threshold was *p*-value 0.01, only SigF and SigG matched the sequence; when the threshold was *p*-value 0.05, SigL, CcpA, Mta, CodY, Xre, AhrC, SigH, SigF, and SigG were included. Therefore, SigF and SigG have the possibility to regulate the expression of *bvs196*, and the promoter regions of SigF and SigG were 4 nt upstream of the *bvs196* putative transcription start site. TransTermHP found a putative rho-independent transcription terminator in the 3′ end sequences of *bvs196* (Figure 1C). *bvs196* was located in a 490-bp intergenic region between *hinT*, HIT family protein, and *ecsA*, encoding ABC transporter ATP-binding protein. We searched the orthologous regions of the *bvs196* via BLASTn, and found them in an impressive evolutionary conservation phylogeny tree consisting of 253 *Bacillus* genomes (including *B. velezensis* strain PEBA20). It was found, by comparative analysis of the genomic context, to be similarly conserved in the genomic region among all 253 genomes of *bvs196* of *B. velezensis* strain PEBA20 with the other 252 genomes; parameters were E-value ≤ 1 × 10^−10^, query coverage ≥ 75%, and identity ≥ 50%, and Organism Optional was *Bacilli* (taxid:91061), which further illustrated the evolutionary conservation of *bvs196*. Moreover, these 253 genomes are divided into 11 putative sister clades based on a phylogenetic tree (Figure 1D). Within these 253 genomes, the 3′ end sequences of the *bvs196* orthologs are conserved with missing of the 5′ end sequences in *Bacillus sonorensis*, *Bacillus glycinifermentans*, *Bacillus paralicheniformis*, and *Bacillus licheniformis* genomes, in which Query cover is lower than 60% and evolutionary relationships are farther apart than the other *Bacillus* species. 

**Figure 2:**
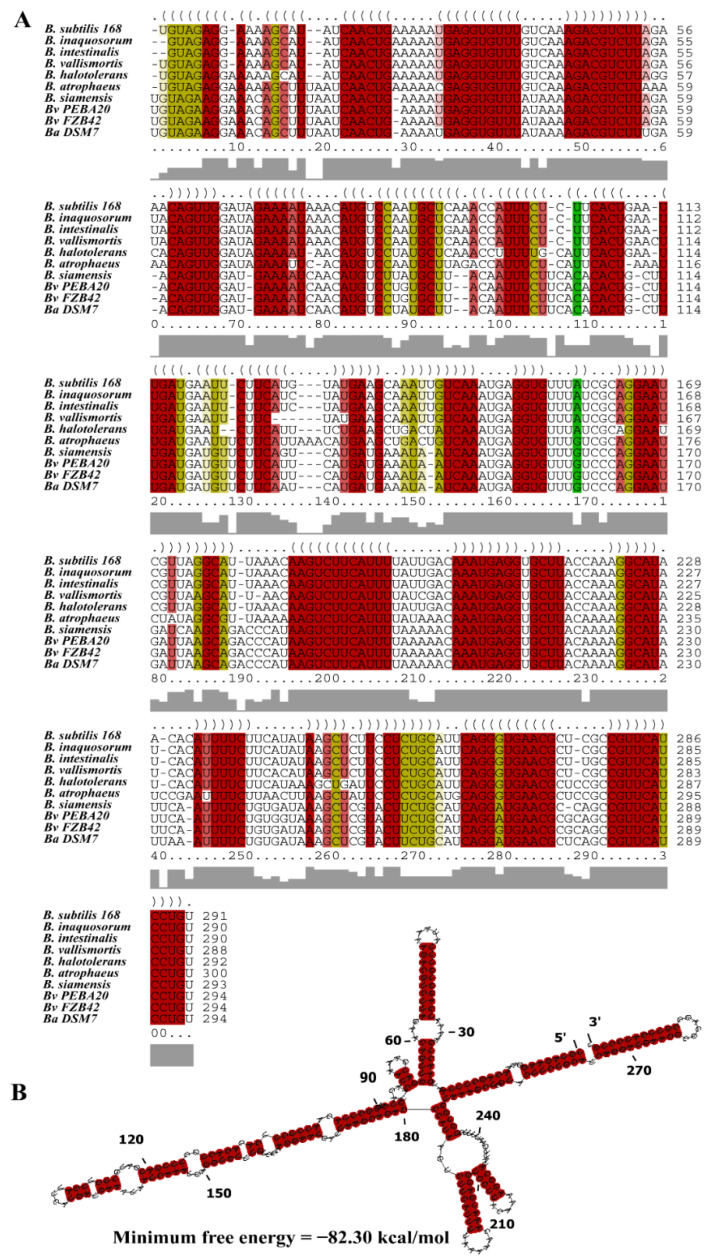
The conservative sequence and secondary structure of *bvs196*. (**A**) LocARNA tool was used to conduct multiple sequence alignment of 10 *bvs196* orthologs. The structural conservation is shown as the different colors: red indicates 100% identical and brown and green indicate one or two nucleotides changed, separately. Bv represents *B. velezensis* and Ba represents *B. amyloliquefaciens*. (**B**) Secondary structure was predicted by RNAalifold, and the minimum free energy value is shown.

A multiple alignment of *bvs196* and its homologs from the other nine *Bacillus* genomes in seven clades was performed using the LocARNA software [26]; the results of the consensus structure indicated that the sequences of stems had high conservation while loops were variable (Figure 2A). This is perhaps due to different interaction sites in the various species. The structures predicted by both LocARNA and RNAalifold [27] were identical, and minimum free energy for the structure was −82.30 kcal/mol which suggested a stable secondary structure, as shown in Figure 2B. These results suggest that Bvs196 very likely acts as a significant conservative regulator in the spore-formation process.

### 3.2. Bvs196 Is a Forespore-Specific sRNA

In order to visualize when and where sRNA Bv196 is expressed in *B. velezensis*, we constructed a transcriptional fusion reporter with Bv196 native promoter to the reporter *gfpmut3a* gene (mutant version of the green fluorescent protein). The transcriptional fusion sequences contain 38-bp downstream regions of the predicted transcription start site and adjunction of the strong ribosome-binding site (AAAGGAGGTGAAATGTACAC).

**Figure 3:**
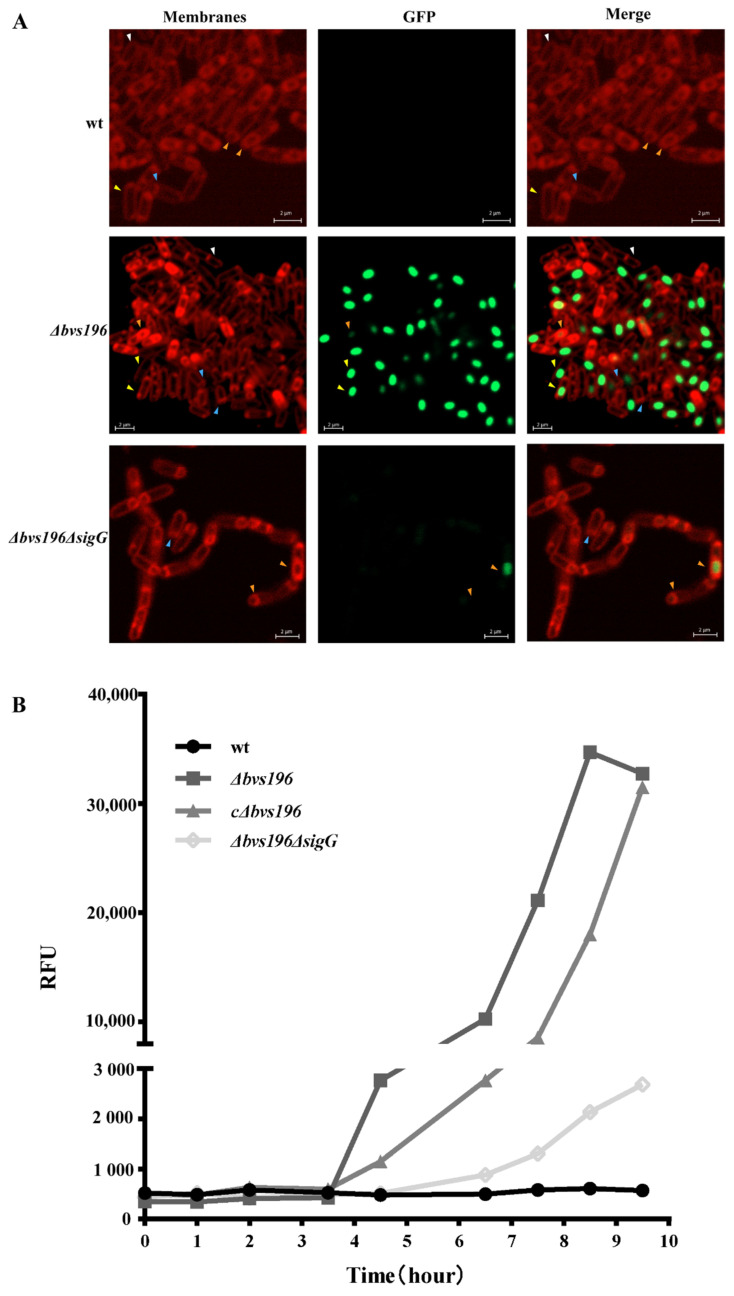
Expression of *bvs196* was controlled by σ^F^ and σ^G^ in the forespore during sporulation. (**A**) Representative fluorescence microscopy images of wt (5 h), *∆bvs196* (6 h), and *∆bvs196ΔsigG* (6 h) mutants at 5 or 6 h after onset of sporulation. Bvs196 activity was monitored by P*_bvs196_-gfp*. Green fluorescence represents the expression of GFP. Red fluorescence from the dye FM4–64 represents the membrane. The FM4–64 dye can label the double membranes between the mother cell and forespore during asymmetric division, but cannot label the membranes after the engulfment. White arrowheads indicate vegetative cells, blue arrowheads indicate cells during polar cell division, orange arrowheads indicate cells during engulfment, yellow arrowheads indicate cells completed engulfment. The scale bar indicates 2 μm. (**B**) Relative fluorescence unit normalized to cell density (RFU) in wt, *∆bvs196*, *∆bvs196ΔsigG*, and *c∆bvs196* during sporulation. Each experiment was repeated three times. Averages from triplicates of time-course expression of a transcriptional P*_bvs196_-gfp* fusion in various strains are shown, with wt as a negative control. Cells were grown in RM medium. Hours of sporulation are indicated.

We monitored the P*_bvs196_*-*gfp* reporter gene of single cells (both vegetative cells and sporulation cells) by fluorescence microscopy. As shown in Figure 3A, we found GFP fluorescence significantly above the background only in the forespores after the completion of engulfment, and there was no GFP fluorescence discovered in any mother cells or vegetative cells. Otherwise, faint GFP fluorescence was observed only in the forespores before the completion of engulfment in *Δbvs196ΔsigG* mutant and *Δbvs196*, and we also discovered that the *Δbvs196ΔsigG* mutant could not complete engulfment [35]. Therefore, deleted *sigG* causes Bvs196 activity to decrease in the forespore leading to inability to complete engulfment. Together, the above demonstrated that a promoter controlled by both σ^F^ and σ^G^ (mainly by σ^G^) is located in the upstream regions of *bvs196*, and the expression of *bvs196* is restricted to the forespore during sporulation.

To further confirm that *bvs196* was controlled by both σ^F^ and σ^G^ (mainly by σ^G^), we quantified the expression levels of *bvs196* during sporulation; the multi-function microplate reader was used to measure the relative fluorescence intensity of various strains in RM liquid sporulation medium during sporulation. As shown in Figure 3B, the relative fluorescence intensity of *Δbvs196* was obviously stronger than the wt background starting at 3.5–4.5 h after the onset of the sporulation and continuously increasing to 8.5 h. During these times, cells were primarily undergoing sporulation stages III, IV, V, and VI. Fluorescence values were not up to the wt background until 6.5 h in *Δbvs196ΔsigG* and were reduced by more than 12-fold compared with *Δbvs196.* This indicated that σ^F^ -directed transcriptional expression of P*_bvs196_* promoter at a low level was not observed immediately and transcriptional level controlled by σ^G^ was significantly upregulated. The fluorescence value in *cΔbvs196* was consistently lower than in *Δbvs196* as part of σ^F^ or σ^G^ was recruited to truncated P*_bvs196_* in *cΔbvs196*. Overall, these results were consistent with the observation of fluorescence microscopy.

### 3.3. Bvs196 Contributes to Resistance of PEBA20

**Figure 4:**
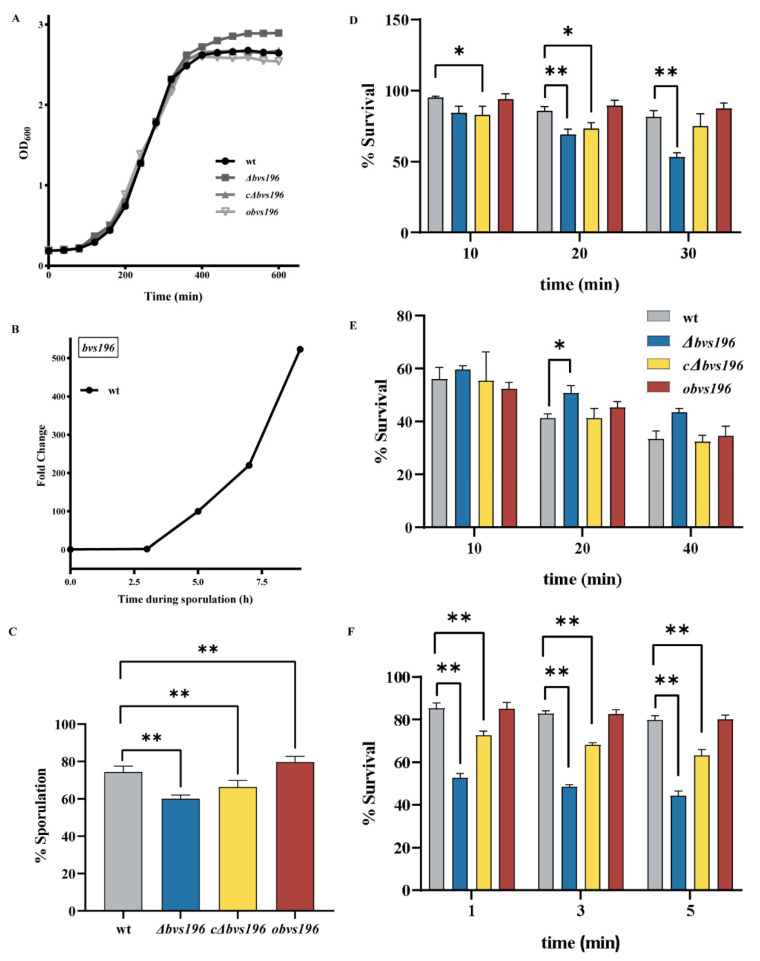
Transcriptional level of *bvs196* and knock-out of *bvs196* led to change in resistance in *B. velezensis* strain PEBA20. (**A**) Growth curves of wild-type strain wt, *Δbvs196*, *cΔbvs196*, and *obvs196* grown on LB. Each experiment was repeated three times. Averages from triplicates are shown. The OD_600_ was measured every 20 min. (**B**) Temporal transcriptional expression of *bvs196* during sporulation in wt. qPCR was used to calculate the relative expression levels of *bvs196* from wild-type (wt)-strain-induced sporulation by RM medium at 37 °C. Time shown is hours after resuspension in RM medium, and the relative expression levels are represented as the fold change to wt at t = 0. Data shown is averages from triplicates. (**C**) Sporulation frequency of wt, *bvs196*, *cΔbvs196*, and *obvs196* at t = 30 h after growing in DSM medium. The sporulation frequency was calculated as colony-forming units/mL (CFUs/mL) after wet-heat treatment divided by CFUs/mL before wet-heat treatment; wet-heat treatment was 20 min at 80 °C. (**D**) The effects of Bvs196 on wet-heat resistance. Spores of each strain were obtained from DSM medium for 60 h and colonies were counted by serial dilution gradient. Spores were treated with wet heat at 85 °C for 0, 10, 20, and 30 min, and the survival percentage was calculated as CFUs/mL after wet-heat treatment divided by CFUs/mL before wet-heat treatment. (**E**) The effects of Bvs196 on formaldehyde resistance. Spores were treated with 2.5% formaldehyde for 0, 10, 20, and 40 min, and the survival percentage was calculated as CFUs/mL after formaldehyde treatment divided by CFUs/mL before formaldehyde treatment. (**F**) The effects of Bvs196 on UV resistance. Spores were treated with UV for 0, 1, 3, or 5 min, and the survival percentage was calculated as CFUs/mL after UV treatment divided by CFUs/mL before UV treatment. Bar graphs represent the average percentages from three independent replicates, and error bars represent standard deviations. Statistical analyses were carried out using DPS data processing system 9.01 software. Analysis of variance (ANOVA) was performed between treatments. *p*-value ≤ 0.05 was considered significant. * *p* < 0.05, ** *p* < 0.01.

It was demonstrated above that *bvs196* is highly conserved among *Bacillus* species and *bvs196* is weakly controlled by early forespore-specific sigma factor σ^F^ and strongly controlled by late forespore-specific sigma factor σ^G^ in the prespore. Hence, we speculate that Bvs196 maybe have a widely conserved function in sporulation. Therefore, to investigate the contribution of Bvs196 in *B. velezensis*, we completely deleted *bvs196* via homologous recombination and constructed plasmid-based complementation strain *cΔbvs196* and overexpression strain *obvs196*. As shown in Figure 4A, the growth of *Δbvs196*, *cΔbvs196*, and *obvs196* showed no significant difference compared with wt. RT-qPCR analysis was used to examine *bvs196* expression at the different stages during sporulation. As shown in Figure 4B, Bvs196 was accumulated in wt cells during the spore formation process, and no expression of *bvs196* was detected in the *Δbvs196* cells during the sporulation. These results were consistent with the results of Figure 3B. Notably, Bvs196 was transcribed with a very low level (1.88 foldchange) at 3 h in sporulation compared to t = 0 h. Then, Bvs196 was upregulated to 53.08 foldchange at t = 5 h compared to t = 3 h, 2.20 foldchange at t = 7 h to t = 5 h, and 2.38 foldchange when at t = 9 h to t = 7 h.

It is known from fluorescence microscopy that the *Δbvs196* strain has no spore formation defect. In order to further understand the role of Bvs196 in *B. velezensis* during sporulation, we first investigated if lacking *bvs196* influences the process of sporulation by measuring the sporulation frequencies of wt, *bvs196*, *cΔbvs196*, and *obvs196*. We found that changes in the expression levels of *bvs196* led to changes in sporulation frequency, with deleted *bvs196* decreasing the sporulation frequency and overexpressed *bvs196* increasing the sporulation frequency, suggesting that Bvs196 regulates the process of sporulation through an unknown regulatory pathway (Figure 4C). The effect of lack of *bvs196* could be partly compensated by overexpression of wild-type *bvs196* from pHTCE, in which *bvs196* was ectopically expressed, indicating that the location of chromosome was very essential. Secondly, we detected if the mature spores isolated from the mutant strains were more sensitive to heat (Figure 4D), formaldehyde exposure (Figure 4E), and ultraviolet (UV) radiation (Figure 4F). Subsequently, we found that *∆bvs196* always caused less resistance to these stresses (except for formaldehyde exposure), suggesting that Bvs196 affects the resistance properties of *B. velezensis* spores by post-transcriptional regulation of resistance-related gene expression.

### 3.4. spo0A and sspN-tlp mRNA Are Direct Targets of Bvs196

**Figure 5:**
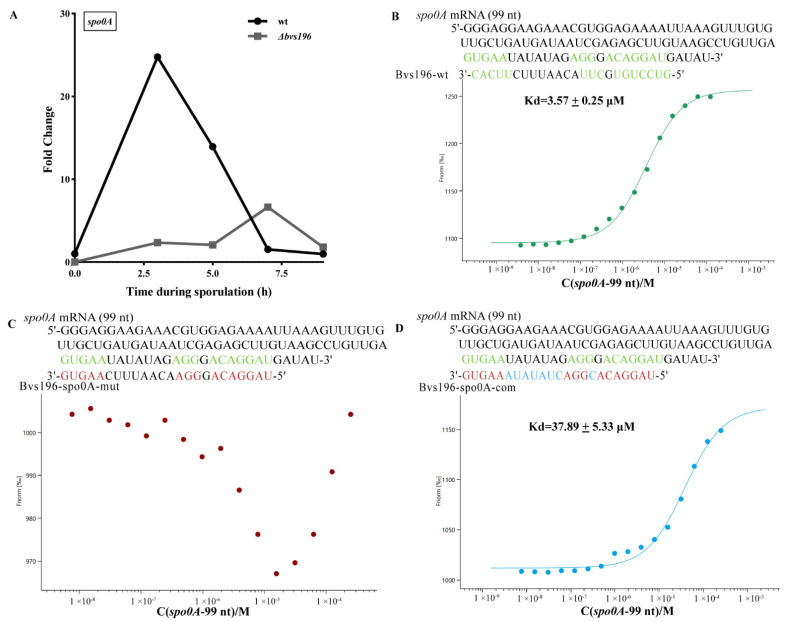
Transcriptional level of *spo0A* and the binding affinity of sRNA Bvs196 with *spo0A* by MST analysis in vitro. (**A**) temporal transcriptional expression of *spo0A* during sporulation in wt and *Δbvs196*. qPCR was used to calculate the relative expression levels of *spo0A* from wild-type (wt)- and *Δbvs196* strain-induced sporulation by RM medium at 37 °C. Time shown is hours after resuspension in RM medium, and the relative expression levels are represented as the fold change to wt at t = 0. Data shown is averages from triplicates. (**B**–**D**) Paired bases are shown in green. Point mutations are shown in red and blue. The Kd value reflects the binding affinity between two molecules; wt indicates wild type; mut indicates mismatch mutation; com indicates compensatory mutation. (**B**) *spo0A* mRNA (99 nt)/Bvs196-wt, the green curve; (**C**) *spo0A* mRNA (99 nt)/Bvs196-spo0A-mut, the red curve; and (**D**) *spo0A* mRNA (99 nt)/Bvs196-spo0A-com, the blue curve.

**Figure 6:**
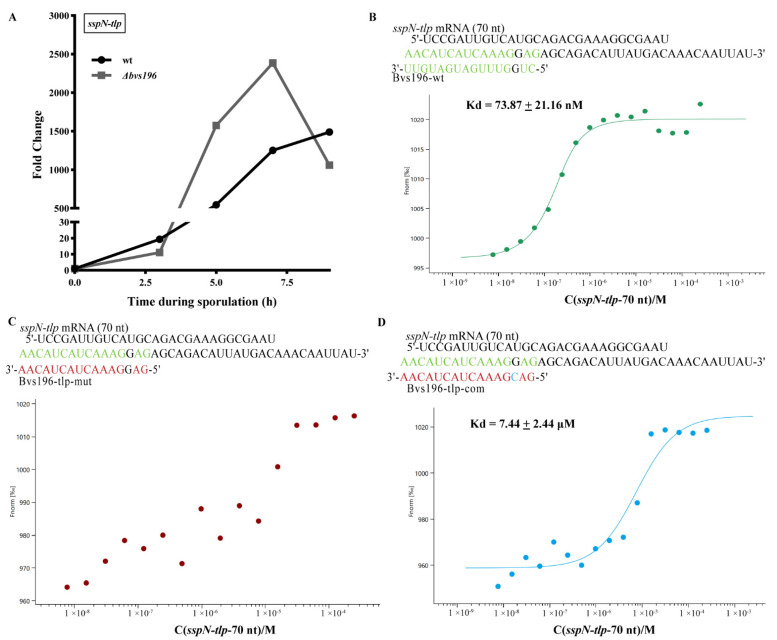
Transcriptional level of sspN-tlp and the binding affinity of sRNA Bvs196 with sspN-tlp by MST analysis in vitro. (**A**) Temporal transcriptional expression of *sspN-tlp* during sporulation in wt and *Δbvs196*. qPCR was used to calculate the relative expression levels of *sspN-tlp* from wild-type (wt)- and *Δbvs196* strain-induced sporulation by RM medium at 37 °C. Time shown is hours after resuspension in RM medium, and the relative expression levels are represented as the fold change to wt at t = 0. Data shown is averages from triplicates. (**B**–**D**) Paired bases are shown in green. Point mutations are shown in red and blue. The Kd value means the binding affinity between two molecules; wt indicates wild type; mut indicates mismatch mutation; com indicates compensatory mutation. (**B**) *sspN-tlp* mRNA (70 nt)/Bvs196-wt, the green curve; (**C**) *sspN-tlp* mRNA (70 nt)/Bvs196 -tlp-mut, the red curve; and (**D**) *sspN-tlp* mRNA (70 nt)/Bvs196-tlp-com, the blue curve.

In order to determine the roles of Bvs196 in spore resistance, we first needed to find the potential targets of Bvs196. Bioinformatic methods, including three web tools TargetRNA2, CopraRNA 2.1.3 and IntaRNA 3.2.0, were used for screening putative mRNA targets of Bvs196. Subsequently, further analysis of the above results was conducted to integrate with operon information into BSGatlas [36] and to apply Inferelator [37]. According to the operon information in BSGatlas, the putative binding sites located inside the genes of operon were excluded. The remaining putative targets were imported to Inferelator and the most likely sRNA–mRNA pairs were recovered. Finally, we selected *spo0A* and *sspN-tlp* mRNAs which were highly likely to interact with Bvs196 and used these to run molecular interaction analysis in vitro Using MST experiments, *spo0A* was predicted by both CopraRNA (rank 120 by *p*-value) and Inferelator (recovered from the priors), and *tlp* was included in both IntaRNA (rank 33 by *p*-value) and TargetRNA2 (rank 3 by *p*-value). Meanwhile, many sporulation-related genes (including *spo0A* and *tlp*) were selected to evaluate the transcriptional levels during sporulation by qRT-PCR (see Figure 5A, Figure 6A and Figure S1).

The computational RNA predictive interaction online tool CopraRNA revealed that Bvs196 (74–131 nt) could bind *spo0A* mRNA (1–59 nt after start codon) with energy of −10.05 kcal/mol, and similarly, Bvs196 (109–124 nt) could bind *sspN-tlp* mRNA (11–26 nt before *tlp* start codon and exactly the intergenic region of the operon) with energy of −13.67 kcal/mol. Interestingly, the binding regions of Bvs196 with the two mRNAs were all located on several overlapping interior loop structures. Therefore, we designed a series of oligonucleotides to test and verify direct interaction between them by MST analysis. As shown in Figure 5, the binding affinity between Bvs196-wt and *spo0A* mRNA was strong with a dissociation constant (Kd) of 3.57 ± 0.25 μM (Figure 5B). Mutation in binding sites of Bvs196 caused no binding affinity between Bvs196-spo0A-mut and *spo0A* mRNA (Figure 5C). As expected, the complementary the non-binding site of Bvs196-spo0A-mut (Bvs196-spo0A-com) could recovery the binding affinity but this was reduced by more than 10-fold (Figure 5D). The binding affinity between Bvs196-wt and *sspN-tlp* mRNA was extremely strong with a dissociation constant (Kd) of 73.87 ± 21.16 nM (Figure 6B) and changing of binding affinity between Bvs196-tlp-mut or Bvs196-tlp-com and *sspN-tlp* mRNA was the same as the mutants of Bvs196 and *spo0A* mRNA, and Bvs196-spo0A-com could recover the binding affinity but this was reduced by more than 100-fold for just one complementary base (Figure 6C,D). All of the above demonstrated that both *spo0A* and *sspN-tlp* mRNAs are in direct interaction with Bvs196 and their binding sites are different, as predicted.

qRT-PCR analysis of the *spo0A* gene indicated that the transcriptional level of *spo0A* in *Δbvs196* was down-regulated in the early stage and up-regulated in the late stage of sporulation compared with wt (Figure 5A), suggesting a complex regulation mode between Bvs196 and *spo0A*. In addition, *sspN-tlp* mRNA levels in *Δbvs196* were up-regulated with a 2.89 foldchange compared to wt at the 5 h after the onset of sporulation, at which time the *bvs196* gene was maximum expression efficiency. This indicated that Bvs196 perhaps negatively affects the expression of *sspN-tlp* genes at a particular stage of sporulation.

## 4. Discussion

The research on the regulation of spore formation has been carried out for several decades, and we clearly understand there is a hierarchical regulatory network of alternative sigma factors and secondary transcription factors, as well as some complex regulatory loops (positive, negative feedback, and feedforward loops) involved [3]. Based on the current research work on sRNAs, it is easy to estimate that many unknown sRNA regulators are present in the regulatory network of sporulation. In 2012, 1583 new RNA fragments were predicted by microarray, and the sigma factor in *Bacillus subtilis* was predicted from all promoter regions. The results showed that the all-independent RNA category contains more sRNAs regulated by alternative sigma factors (included σ^B^, σ^E^, σ^F^, and σ^K^), CsfG, and S547 (Bvs196 homolog). This may indicate that sRNAs might play a more important regulatory role in conditions with stress and sporulation [13]. In 2016, Mars classified 1583 RNA fragments according to evolutionary conservation, predicted secondary structure and expression level, and then predicted their functions by biological context [19]. This further illustrated that the sporulation-related sRNAs, regulated by sporulation sigma factors, are likely to directly or indirectly interact with transcription factors to regulate spore formation and even affect spore resistance, germination, and growth.

Previously, it was confirmed that the spore properties were influenced by the medium conditions during sporulation, and that the quality of spores formed from nutrient-rich conditions was higher than starvation conditions [34]. Bvs196 was detected under various conditions, including rich medium LB, minimal medium M9, N-starvation medium NSM9, biofilm medium, Difco sporulation medium, DSM, and resuspension medium RM. It indicated that Bvs196 may play completely different biological roles in the above culture conditions.

Spo0A is a master regulator and controls the initiation of spore formation, which was proved to directly and indirectly drive expression of hundreds of genes [35,36]. We detected, by qRT-PCR analysis, that transcriptional levels of genes (*spoIIE*, *spoIIB*, *spoIIAA*, *spoIIAB*, *sigF*, *spoIIGA*, and *sigE*) were regulated directly by Spo0A and that most of them were down-regulated by the absence of Bvs196 (Supplementary Figure S1), which also confirmed the positive role of Bvs196 in the control of *spo0A* at the early stage of sporulation. However, what is confusing is that previous studies have demonstrated that Spo0A is only at high levels in the mother cell after asymmetric division [37]—the time that Bvs196 expression is low and under the control of SigF. The question is, how does Bvs196 interact with the *spo0A* mRNA? Bvs196 could cross the SpoIIIA-SpoIIQ feeding tube to the mother cell or Bvs196 may be positively regulating *spo0A* mRNA for a short time until Bvs196 is controlled by SigG, then, maybe, Bvs196 negatively acts on *spo0A* mRNA after completing engulfment. Just as in the control mode of Spo0A-P (divided into high- and low-threshold regulons), this could explain the wave 7 h after the onset of sporulation.

A series of small, acid-soluble spore proteins (SASPs) exist in the dormant spores of *B. subtilis*, but among them α, β, and γ-type SASPs compose the majority of all SASPs in spores [38,39]. In addition, a group of minor SASPs were also detected in *B. subtilis* spores [38,40,41,42], the functionality of most of these has not been proven and most of them were the abundant spore mRNAs for unknown reasons [43]. However, researchers believe that these minor SASPs maybe also affect spore properties. SASPs could protect DNA against wet-heat, UV, and damage by some chemicals (including formaldehyde), thereby enhancing the resistance of spores [44]. Mutations of deleted the *sspN-tlp*, encoded two minor SASPs, sporulated well, and exhibited wet-heat and UV resistance [42]. In our study, *sspN-tlp* mRNA was upregulated in *Δbvs196* compared to wt and followed the reduction of wet-heat and UV resistances. This suggests that increasing the amount of *sspN-tlp* mRNA may decrease the other SASPs, particularly the major α/β-type SASPs.

Sporulation is a temporal expression process that follows the turning on or off of some genes by alternative sigma factors and secondary transcription factors. Based on the timing of *bvs196* expression and two targets that were proved above, we make a bold assumption that Bvs196 regulates two group targets that are dependent on the expression level. During pre-engulfment stages, Bv196 expression by SigF is low and always promotes translation or maintains mRNA stability of the target genes (such as *spo0A*). In post-engulfment stages, Bvs196 is very highly expressed by SigG, and could either promote translation or maintain mRNA stability of the target genes (such as *sspN-tlp*) or inhibit translation and promote mRNA degradation (such as genes directed by SigF and its cooperative transcription factors, which are unnecessary in the late stages of sporulation in the forespore). This is an interesting point to be investigated in the future.

Furthermore, different phenotypic assays demonstrated that Bvs196 not only regulates sporulation frequencies but also impacts resistance to heat, formaldehyde, and UV, since Bvs196 may be involved in the trade-off between spore quantity and quality and contributes to the evolutionary adaptation of spore-forming bacteria [45]. Therefore, we hypothesize that Bvs196 adjusts the post-transcriptional expression levels of many genes to ensure efficient utilization of nutrients and avoid energy waste during spore formation for the generation of high-quality spores. Further research will focus on the identification of other Bvs196 targets and their regulation mechanisms. It will also investigate whether the absence of Bvs196 affects the protein composition of spores as well as germination and growth.

## Data Availability

Not applicable.

## Supplementary Materials

The following supporting information can be downloaded at: https://www.mdpi.com/article/10.3390/microorganisms10051015/s1, Table S1: Strains and plasmids used in this study; Table S2: Primers used in this study; Figure S1: Temporal transcriptional expression of *spoIIE*, *spoIIB*, *spoIIAA*, *spoIIAB*, *sigF*, *spoIIGA*, and *sigE* during sporulation in wt and *Δbvs196*.
