# Supplementary material for: A Sporulation-Specific sRNA Bvs196 Contributing to the Developing Spore in Bacillus velezensis
Source: Microorganisms. 2022 May 12;10(5):1015. doi: 10.3390/microorganisms10051015 (PMC9147052; doi:10.3390/microorganisms10051015)
Supplement: Supplementary file 1 [file microorganisms-10-01015-s001.zip › microorganisms-1713911-supplementary.pdf]

Supplementary Materials

A Sporulation-Specific sRNA Bvs196 Contributing to the Developing Spore in *Bacillus velezensis*

1. Supplementary Tables

Table S1. Strains and plasmids used in this study.

Strain, plasmid	Genotype and phenotype	Source
***B. velezensis* strains**		
PEBA20	*B. velezensis* PEBA20, wild type	Laboratory stock
Δbvs196	PEBA20, *Δbvs196* :: *gfpmut3a*	This study
cΔbvs196	*Δbvs196*, pHTCE-Bvs196, Cm^r	This study
obvs196	PEBA20, pHTCE-Bvs196, Cm^r	This study
ΔsigG	PEBA20, *ΔsigG*	This study
Δbvs196ΔsigG	*Δbvs196*, *ΔsigG*	This study
**Plasmid**		
pMAD	Amp^r in *E. coli*, Em^r in *Bacillus*	This study
pHT01	Amp^r in *E. coli*, Cm^r in *Bacillus*	This study
pHTCE	Derivative of pHT01with no P*grac*, Amp^r in *E. coli*, Cm^r in *Bacillus*	This study
pHTCE-*bvs196*	pHTCE contained *bvs196* with its native promoter, Amp^r in *E. coli*, Cm^r in *Bacillus*	This study

Table S2. Primers used in this study.

Primer Name	Sequences (5'–3')	Purpose
PMAD_gfpmut 3-R	CTGATGCAGAAGTACGAGCTTTATTTGTATAGTTCATCCATGCCATG	*Δbvs196* deletion mutant consturction
PMAD_gfpmut 3_F	GAAACAGCTTAAAGGAGGTGAAATGTACACATGCGTAAAGGAGAA GAA	
pMAD_Bvs196 UP_F	ATCTATCGATGCATGCCATGGTGTCTTTGACCCACTTGCTGTACC	
pMAD_Bvs196 UP_R	CACCTCCTTTAAGCTGTTTCCTTCTACATACATTATGA	
pMAD_Bvs196 down_F	AGCTCGTACTTCTGCATCAGGAT	
pMAD_Bvs196	GCGTCGGGCGATATCGGATCCAATCGTCGGGAATATCCATCAG	

down_R		
sigG-up-F	ATCTATCGATGCATGCCATGGGCGTCTGACATATTTGTGGTATAAATG	Δ*sigG* deletion mutant consturction
sigG-up-R	CTTGGGCCTCATCCTGTAATTGCCTAAACA	
sigG-down-F	ATTACAGGATGAGGCCCAAGCTCAGGTCTCAAGGC	
sigG-down-R	GCGTCGGGCGATATCGGATCCTTTACGGATGATGCCTCTTTTGC	
pHTCE_F	TCCTGCAGTCTAGACTCGAGAGATCTTTGTCTGCAACTGAAAAGT	pHTCE plasmid consturction
pHTCE_R	GGAGCTCGGTACCGACGTCTTTTTAATGTGGTCTTTTATTCTTC	
pHTCE_Bv196_F	AGACGTCGGTACCGAGCTCCTTTCTCCCCTTCTGATTCGTTTT	pHTCE-*bvs196* plasmid consturction
pHTCE_Bv196_R	CTCGAGTCTAGACTGCAGGAAGATGGGTTTCCTCCCTGAAA	
Bvs196	GACGTCTTAGAACAGTTGGATG	northern blot
	TAATACGACTCACTATAGGGAGAGTTCATCCTGATGCAGAAGTAC	
5S	GTGGCGATAGCGAAGAGGTCA	
	TAATACGACTCACTATAGGGAGATTGGCGGCGTCCTACTCTCA	
bvs196--F	TAATACGACTCACTATAGGGAGAGGCGGACATCATAATGTATGTAG	MST
bvs196-R	ACAGGATGAACGGCTGCG	
bvs196-sspN-tlp-mut-F	AgaggaaactactacaaCTTCATTCATGATGAAATAATCAAATGA	
bvs196-sspN-tlp-mut-R	GttgtagtagtttcctcTGTGTGAAGAAATTGTAAGCACAGG	
bvs196-sspN-tlp-com-F	AgacgaaactactacaaCTTCATTCATGATGAAATAATCAAATGA	
bvs196-sspN-tlp-com-R	GttgtagtagtttcgtcTGTGTGAAGAAATTGTAAGCACAGG	
bvs196-spo0A-mut-F	CATCAggacaggaaacaatttcaagtgACACTGCTTTGATGATGTTCTTCAT	
bvs196-spo0A-mut-R	tgaaattgtttcctgtcctgATGTTGATTTTCATCCAACTGTTCTAA	

bvs196-spo0A-com-F	CATCAggacacgaatgttaaagaagtgACACTGCTTTGATGATGTTCTTCAT	
bvs196-spo0A-com-R	tctttaacattcgtgtcctgATGTTGATTTTCATCCAACTGTTCTAA	
spo0A_mRNA(99 nt)	GGGAGGAAGAAACGUGGAGAAAAUUAAAGUUUGUG UUGCUGAUGAUAAUCGAGAGCUUGUAAGCCUGUUGA GUGAAUAUAUAGAGGGACAGGAUGAUAU	
sspN-tlp_mRNA(70 nt)	UCCGAUUGUCAUGCAGACGAAAGGCGAAUAACAUCAUCAAAGGAGAG CAGACAUUAUGACAAACAAUUAU	
Bv196-F	GGTGTTTGTCCCAGGAATGAT	qRT-PCR
Bv196-R	ATCCTGATGCAGAAGTACGAGC	
spoIIE-F	GGACGAACTTGCCCACCAT	
spoIIE-R	CCGCCATGACCTCAGAAAC	
spo0A-F	GCTTGTAAGCCTGTTGAGTGAAT	
spo0A-R	AACCGCAAGTCCGTCCAGAT	
spoIIB-F	CCTCCGCTCCGACATCA	
spoIIB-R	CAAGCGTTTGCGCTCCTT	
sigF-F	TTGATGCGGAGGATGTGGT	
sigF-R	AAGCAGGGTGATCGGGTCT	
spoIIAB-F	ACGCTGGAGGATCATGTCG	
spoIIAB-R	GCGCTCAAGCTCGGGTTTT	
spoIIAA-F	GGTTACTGAATCGCTGGAGG	
spoIIAA-R	CGCCGAGCTGTTTAATTTGT	
spoIIGA-F	GCGAAAGGATTGGTGGATT	
spoIIGA-R	CTGGCGGATTTGATGAGGT	
sigE-F	TCAAACTCGCCACCTACGC	
sigE-R	GCTCGTTTCCGTCCCAATC	
tlp-F	CAATGGGATTTGCGACAGA	
tlp-R	CGATTCATCCTGGATTTCACT	
16S rDNA-F	GGAGGCAGCAGTAGGGAATCTT	

16S rDNA-R	GCCGTGGCTTTCTGGTTAGGT

2. Supplementary Figure

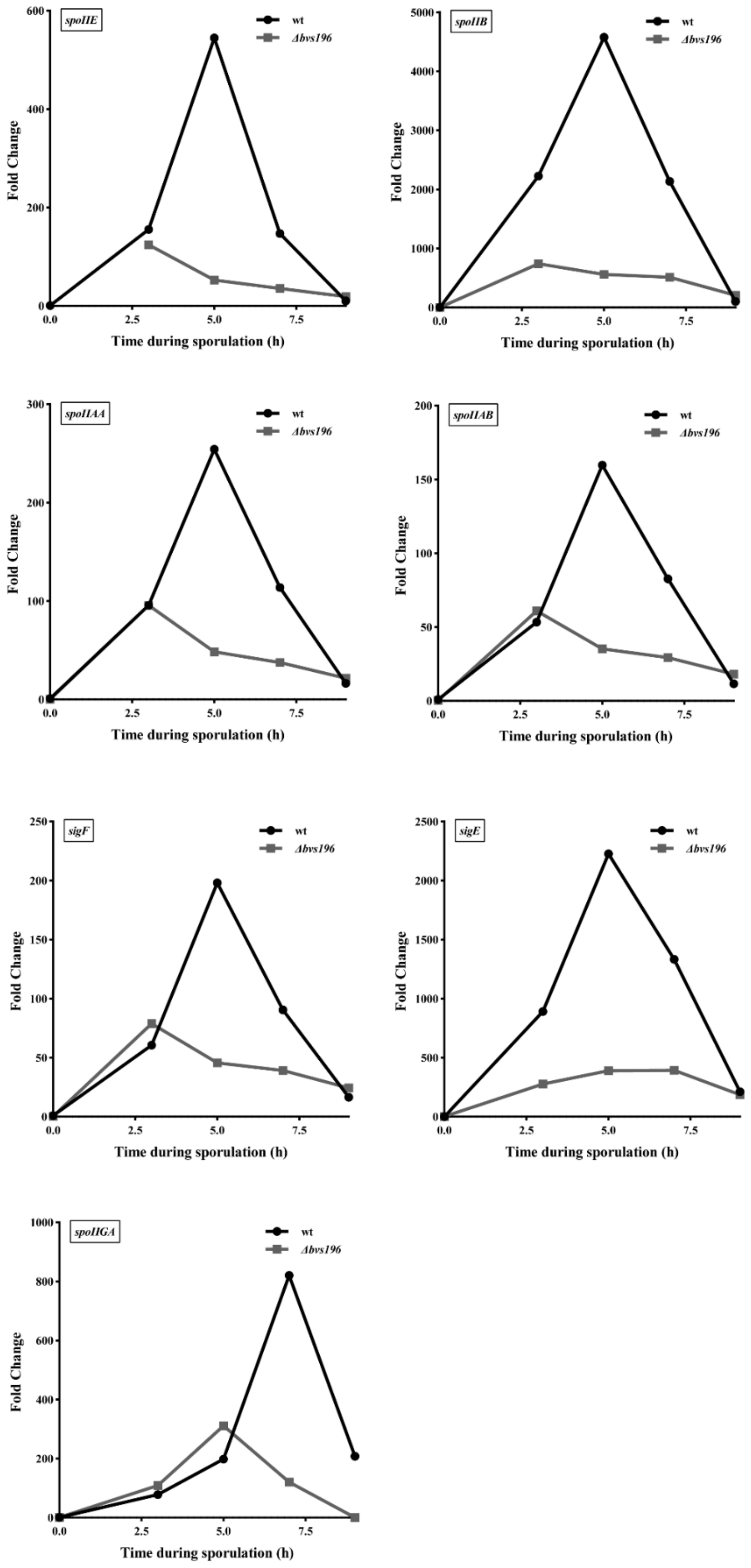

Figure S1. temporal transcriptional expression of *spoIIE*, *spoIIB*, *spoIIAA*, *spoIIAB*, *sigF*, *spoIIGA* and *sigE* during sporulation in wt and *Δbvs196*. qPCR was used to measure relative mRNA levels of *spoIIE*, *spoIIB*, *spoIIAA*, *spoIIAB*, *sigF*, *spoIIGA* and *sigE* from wild-type (wt) cells and *Δbvs196* cells grown at 37°C in RM medium. Time shown is hours after resuspended in RM medium, and mRNA levels are given as the fold change relative to wild-type levels at t = 0. Data shown are from one representative experiment with three replicates.
